# Retroperitoneal Abscess Formation Extending to the Lumbar Area After Extracorporeal Shock Wave Lithotripsy

**DOI:** 10.1089/cren.2018.0072

**Published:** 2018-12-21

**Authors:** Michael Nomikos, Anastasios Tsalavoutas, Angelika Kalpaxi, George Athanasopoulos

**Affiliations:** ^1^ Department of Urology, Thriassion General Hospital, Athens, Greece.; ^2^ Department of Radiology, Thriassion General Hospital, Athens, Greece.

**Keywords:** retroperitoneal abscess, shock wave lithotripsy, nephrectomy

## Abstract

***Background:*** Renal rupture and retroperitoneal abscess formation after extracorporeal shock wave lithotripsy (SWL) is a rare and potentially life-threatening complication if left untreated with a high morbidity rate. In this study, we present a rare case of renal rupture after SWL, with formation of an extensive retroperitoneal abscess extending to the left abdominal and lumbar area.

***Case Presentation:*** A 48-year-old Caucasian woman presented to the outpatient department with left abdominal and lumbar redness and swelling caused by renal rupture and massive perinephric abscess formation, 10 days after SWL treatment of her left renal pelvic stones. She was treated first with drainage of the retroperitoneal abscess and simultaneous Double-J stent placement in her left kidney. A left open nephrectomy was subsequently performed because of persistence of kidney infection.

***Conclusion:*** Retroperitoneal abscess formation after SWL is a serious and highly morbid complication, which should be early diagnosed and timely treated.

## Introduction and Background

Extracorporeal shockwave lithotripsy is an established treatment modality for the past 35 years in the management of urinary stone disease. The majority of complications that arise are usually minor and well tolerated, but the accumulated experience has shown that severe and highly morbid conditions can also occur. Infectious complications can arise as a result of traumatizing effect on renal parenchyma and infected residual stone fragments, which can promote bacteriuria, perinephric abscess formation, and urosepsis. A perinephric abscess is a collection of suppurative material in the perinephric space, which is a result of renal collective system rupture after extracorporeal shock wave lithotripsy (SWL).^1^ A perinephric abscess should be suspected when fever and flank pain occur a few days after SWL. A delay in diagnosis could significantly increase the morbidity. In this study, we present a case of retroperitoneal abscess formation after SWL, extending to the ipsilateral lumbar area.

## Case Presentation

**FIG. 1.**
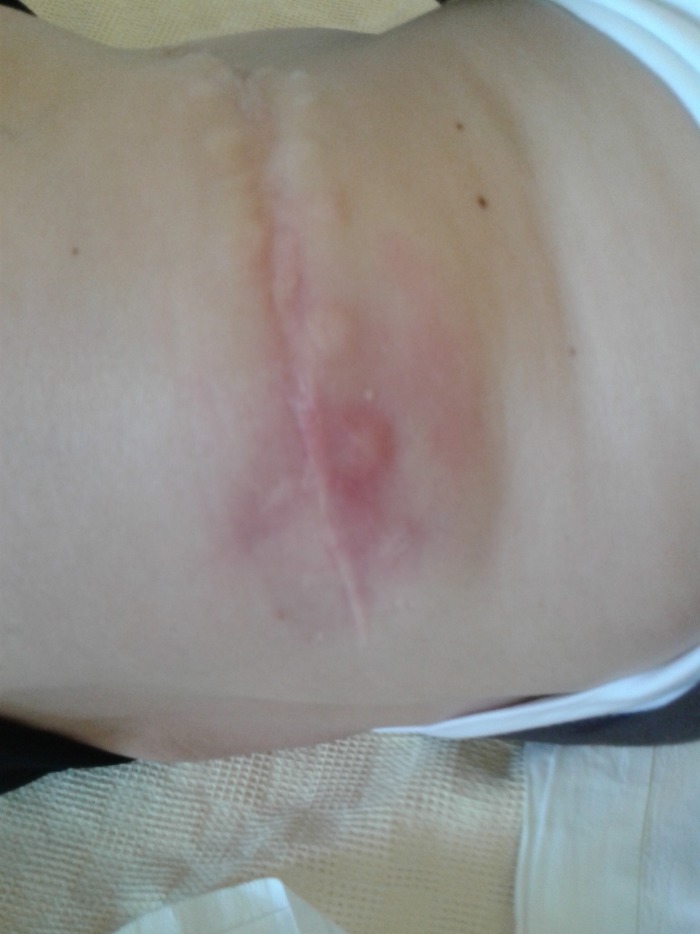
Redness and swelling of the left abdominal and lumbar area.

**FIG. 2.**
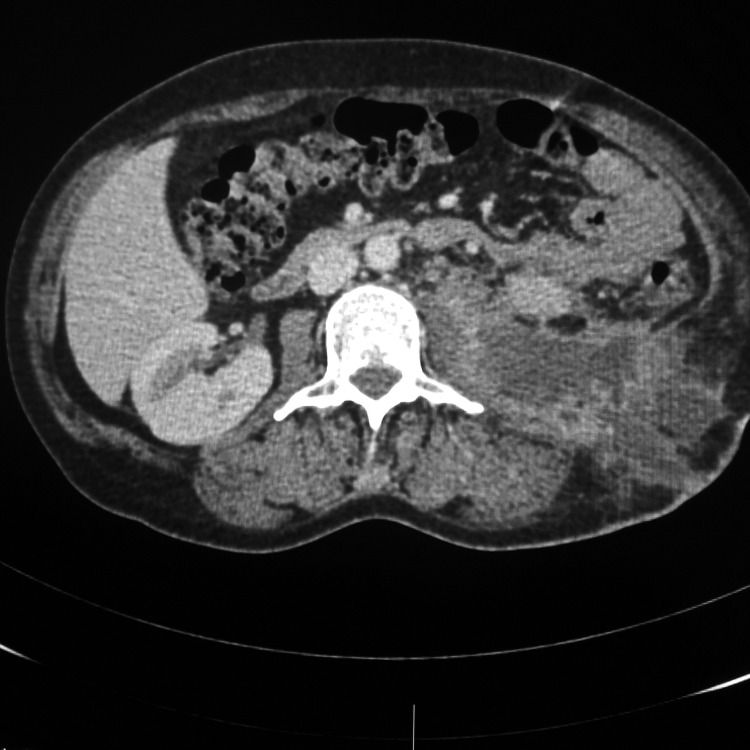
Sagittal section of CT of the abdomen showing large retroperitoneal and subcutaneous pus collection arising from the left kidney.

**FIG. 3.**
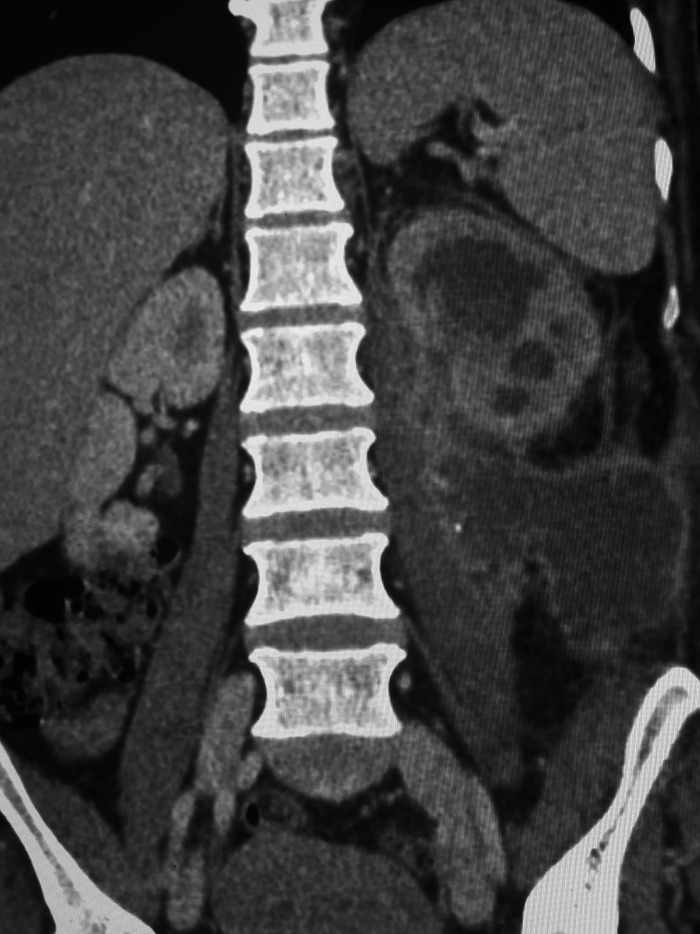
Coronal section of CT of the abdomen showing large retroperitoneal collection around the left kidney infiltrating the ipsilateral psoas muscle.

A 48-year-old Caucasian woman presented in the outpatient department with a 1-week history of pain, redness, and swelling in the left abdominal and lumbar area after an SWL treatment for her left renal stones 10 days before her presentation. Urine culture before SWL was sterile. Clinical examination on arrival showed rising swelling and redness extending from left lumbar to left abdominal area (Fig. 1). The patient was hemodynamically stable and afebrile. She reported two pyelolithotomies in each kidney during her adolescence. Her full blood count, urea, and creatinine values were unremarkable. Her C-reactive protein levels were elevated at 40.90 mg/L. Urinary culture received on arrival was sterile, probably because of a 7-day course of ciprofloxacin after SWL. The CT scan of the abdomen with intravenous contrast revealed hydronephrosis and multiple stones in the left renal pelvis, as well as fluid attenuation of ∼30 HU-suggesting of pus within the left perirenal and pararenal area; the psoas muscle was also infiltrated and the collection extended toward the skin (Figs. 2 and 3).

**FIG. 4.**
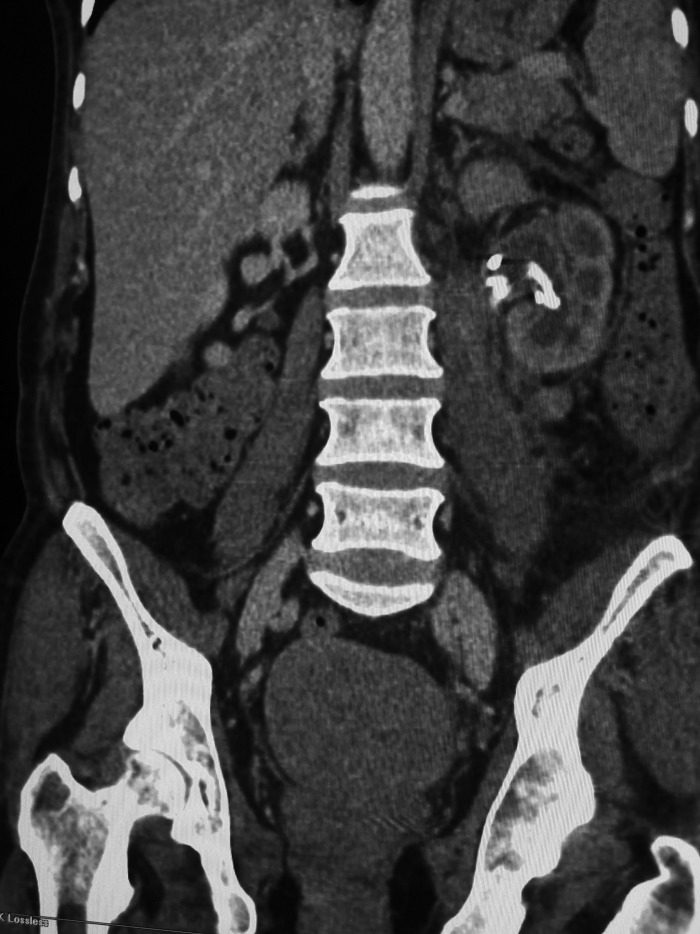
CT of the abdomen 4 days after surgical debridement, showing significant reduction of the collection with a Double-J stent in the left renal pelvis.

Piperacillin/tazobactam and clindamycin were immediately administered and the patient was taken to the operating room. She was placed in the Galdakao-modified supine Valdivia position and a 7F/28 cm Double-J (D-J) stent was inserted. A 5 cm incision was afterward made on the swelling of the left lumbar area and 400 mL of pus was drained from the subcutaneous, retroperitoneal, and psoas muscle area. Two drains were placed in the perirenal space. The culture of pus was sterile. The drains were removed on the fourth postoperative day and the patient remained afebrile. On the fifth postoperative day she developed fever, tachycardia, and leukocytosis (white blood cells 22,500). A new CT scan was performed, which showed significant reduction of the perirenal accumulation, with remaining pus in the renal calyces (Fig. 4). Despite the presence of D-J stent, a nephrostomy tube was additionally inserted, which did not produce enough pus. The patient remained septic during the following day (T_max_ = 38.5°C) and an urgent open nephrectomy was performed. Imipenem was then administered. Second pus culture from the renal cavities yielded no pathogens. She had a rapid clinical and laboratory recovery and she was discharged on the sixth postoperative day after nephrectomy. The overall hospitalization period was 12 days. Histopathology revealed necrotic inflammation with abscess in both renal pelvis and parenchyma, multiple histiocytes and ulceration of the epithelium of the pelvis, pelvis rupture and lower pole parenchyma deficit, probably because of the shockwaves.

## Discussion

Since its introduction in clinical practice in 1982, SWL has become the preferred treatment modality for the management of urinary stone disease. Although minimally invasive, severe complications have been reported in the literature.

The formation of an abscess as a complication of SWL is rare with only five cases reported in the literature. SWL gives rise to bacteremia in ∼4% of patients. Bacteria arising from the fragmented stone or entering by way of infected urine are thought to cause abscess formation by lodging in the damaged tissue and extravasated blood or urine.^2^ This complication can occur because of parenchyma disruption of the kidney from the shock waves and rupture of focal intraparenchymal abscess, which leads to spread of infection into the perinephric area with abscess formation. Simultaneous abscess foci in multiple organs after SWL have also been reported.^3^

To our knowledge, this is the first report in the literature of renal rupture and enormous retroperitoneal abscess formation after SWL, spreading to ipsilateral flank and abdominal skin without obvious signs of pyelonephritis before SWL. Possible predisposing factors were pre-existing kidney obstruction and infected stone load.

The mainstay of treatment is early diagnosis with thorough clinical history and physical examination accompanied with CT scan of the abdomen. Prompt management with relief of kidney obstruction and percutaneous drainage of the perirenal collection are suggested in relevant literature for the treatment of such a devastating SWL complication. The supine modified Galdakao Valdivia position used in the first procedure was useful, since it allowed the simultaneous kidney decompression with D-J stent placement and retroperitoneal abscess drainage.^4^

Close monitoring of the patient with open surgical debridement should be attempted in cases that infection persists after percutaneous drainage or in patients with multiloculated, large, or gas formatting perinephric abscesses. Antibiotics help to control sepsis and to prevent the spread of infection. In our case, open nephrectomy was eventually performed because of persistence of urinary sepsis despite percutaneous nephrostomy insertion in the infected already stented kidney.

## Conclusion

SWL, although minimally invasive, is not without complications. Proper patient selection with individual evaluation of preprocedural risk factors with respect to the presence of urinary tract infection and obstruction should be performed to avoid post-SWL devastating infective complications.

## Abbreviations Used

CT: computed tomography
D-J: Double-J
SWL: extracorporeal shock wave lithotripsy
